# Time and costs of the ethics approval process for retrospective studies in Hanover, Germany

**DOI:** 10.3205/000360

**Published:** 2026-06-15

**Authors:** Max Lukas Linderkamp, Efthymios Papazacharias, Philippe Korn, Nils-Claudius Gellrich, Michael-Tobias Neuhaus

**Affiliations:** 1 Department for Oral and Maxillofacial Surgery, Hannover Medical School, Hanover, Germany

**Keywords:** ethics approval process, retrospective studies, economy in medicine, ethics approval

## Abstract

**Purpose:**

Financial pressure on the German healthcare system is rising, and physicians face high pressure in balancing patient care and research. Into this environment comes an ethics approval process for retrospective studies that is becoming more complex and requires a greater volume of documentation. The objective of this study was to first describe and calculate the costs incurred by the ethics approval process.

**Methods:**

Using ChatGPT and Gemini, the required times for preparation, creation, and submission, as well as the time required for the ethics committee to process and vote on ethics approval, were estimated. Costs were calculated based on the newest version of the collective agreement for physicians at German university hospitals (Tarifvertrag für Ärzte an Universitätskliniken).

**Results:**

Time required for submitting adds up to approximately 4–10 hours, and 4.5–8.5 hours for submission control and voting, respectively. Summing up, this amounts to 161.7–388.1 € for submission and 241.6–456.3 € for the ethics committee, respectively. In total, 8.5–18.5 hours are required for ethics approval for a retrospective study, resulting in a total of 403.3–844.4 €.

**Conclusion:**

Ethics approval is of major relevance to ensure good ethical practice and shall therefore not be omitted. Nevertheless, processes can be tightened to facilitate clinical research, free up resources, and save costs.

## Zusammenfassung

**Zielsetzung:**

Der finanzielle Druck auf das deutsche Gesundheitssystem nimmt zu, während Ärztinnen und Ärzte zunehmend gefordert sind, klinische Versorgung und Forschung miteinander zu vereinbaren. Gleichzeitig wird das Ethikgenehmigungsverfahren für retrospektive Studien zunehmend komplexer und erfordert einen steigenden Dokumentationsaufwand. Ziel dieser Studie war es, den zeitlichen und finanziellen Aufwand des Ethikgenehmigungsverfahrens zu beschreiben und zu quantifizieren.

**Methoden:**

Unter Verwendung von ChatGPT und Gemini wurden die benötigten Zeiten für Vorbereitung, Erstellung und Einreichung der Unterlagen sowie die Bearbeitungsdauer durch die Ethikkommission einschließlich Prüfung und Beschlussfassung abgeschätzt. Die Kostenberechnung erfolgte auf Grundlage der aktuellen Fassung des Tarifvertrags für Ärztinnen und Ärzte an Universitätskliniken (TV-Ärzte).

**Ergebnisse:**

Der Zeitaufwand für die Antragstellung beträgt etwa 4–10 Stunden, für Antragsprüfung und Votierung durch die Ethikkommission 4,5–8,5 Stunden. Daraus ergeben sich Kosten von 161,7–388,1 € für die Antragstellung sowie 241,6–456,3 € für die Antragsbearbeitung und -prüfung. Insgesamt werden für das Ethikgenehmigungsverfahren einer retrospektiven Studie 8,5–18,5 Stunden benötigt, was Gesamtkosten von 403,3–844,4 € entspricht.

**Schlussfolgerung:**

Die Ethikgenehmigung ist von zentraler Bedeutung zur Sicherstellung guter wissenschaftlicher Praxis und darf daher nicht entfallen. Dennoch sollten bestehende Prozesse optimiert werden, um klinische Forschung zu erleichtern, Ressourcen freizusetzen und Kosten zu reduzieren.

## Introduction

On the one hand, ethics approval for studies involving patients or their data is mandatory and inevitable. Ethics approval committees should ensure good ethics in scientific practice, preventing seriously flawed and useless science [1]. However, that does cost a lot of time and money, and sometimes, especially in retrospective studies, ethics approval can feel like more of a burden. Even though most studies must be approved by ethics committees, the evaluation of scientific quality by these committees prior to the study does not appear to ensure high-quality research [2].

On the other hand, the German healthcare system is one of the most expensive in the world, spending 12.3% of its Gross Domestic Product (GDP), and is expected to become even more expensive in the future [3]. Only the United States of America uses relatively more of its GDP. Despite being so expensive, it is one of the least efficient, spending more than the Organization for Economic Co-operation and Development (OECD) average, but having just an average OECD life expectancy [4]. Despite significant financial resources, the healthcare system lacks funding for investments, structural renovations, and staffing [5]. Many experts believe the system is on the verge of collapse, while physicians express concerns over deteriorating working conditions and a lack of time for direct patient care [6]. Efficient workflows are particularly essential in university hospitals, where medical staff must teach and conduct research in addition to providing clinical care.

In recent years, legislative changes and digitalization processes have led to a restructuring of the format and requirements for ethics applications at Hannover Medical School (MHH). Since 2^nd^ of January 2025, the required forms are nationwide consistent due to a new procedure implemented by the German Medical Association (Bundesärztekammer) and the Association of Medical Ethics Committees in Germany (Arbeitskreis Medizinischer Ethik-Kommissionen in der Bundesrepublik Deutschland). As a result, there appears to be a steady increase in both the complexity and volume of documentation required for retrospective studies. Comparable anecdotal evidence exists from the United Kingdom, where a restructuring of ethics committees in the late 1990s was intended to reduce administrative hurdles and unnecessary delays, yet ultimately had the opposite effect [7], [8], [9].

This development is particularly striking, considering that an ethics vote for retrospective studies is not mandatory in all cases and that participation in such research typically entails no additional risks or disadvantages for patients. In some countries, retrospective studies do not require ethics committee approval when they do not involve direct human research [10], [11].

In general, there is no discussion about whether ethics approval is required [12]. There is no doubt that ethics approval is mandatory for good scientific practice and to ensure patient rights and safety.

The objective of this study was to determine the current costs associated with the preparation and submission, as well as the review and formal approval, of an ethics application for a retrospective study at MHH.

## Materials and methods

ChatGPT (GPT-5.2, OpenAI, San Francisco, California) and Gemini (Gemini 3, Google, Mountain View, California) were asked to estimate the time required to prepare and submit an ethics approval at the MHH by a resident physician and the time required by the ethics committee to check the submission and vote for the ethics approval for a retrospective study. To obtain a more precise model, the number of required forms was raised by creating a mock case in the MHH ethics portal “ethikPool”. The extent of free-text fields was estimated based on the length of the free-text fields in an already approved ethics application. The more conservative (fewer hours needed) model was used for the calculation, so ChatGPT provided the data for the submission model, whilst Gemini provided the data for the ethics committee costs. 

Based on the models mentioned above, the costs were calculated using the latest version of the collective agreement for physicians at university hospitals (Tarifvertrag für Ärzte an Universitätskliniken, TVÄ, based on a 40-hour week, applicable since 1^st^ of January 2026). The salary based on the TVÄ Ä1,1 and Ä4,1 were used to calculate the physician residents’ and the head of department’s hourly loan, respectively. As the ethics committee of MHH mostly comprises senior consultants, TVÄ salary Ä3,1 was the basis for hourly loan calculation.

The required forms were analyzed for duplicate information, and the duplicates and their total were noted. 

## Results

The example case for the ethics approval process was created on the “ethikPool” platform, which does not distinguish between the various types of retrospective studies. In total, eight forms were required. These were an application for professional/legal consultation §15 (four pages, partly free text, partly multiple-choice questions), a declaration of suitability of the study center (two pages, requires the signature of the head of the department), a list of the participating study centers (length depends on the number of centers, for monocentric studies usually one page), information on project funding and cost coverage statement (two pages), an informal cover letter (length depending on the author and the complexity of the scientific project, at least one page), a study protocol/project plan (free and running text, answering eight key points, signature on a separate page separately uploaded, for example: for a retrospective study ethics application with positive approval: three pages), a structured synopsis of the project plan (non-writable pdf document, at least one page) and a data protection statement featuring a roles and permission concept (no template; free text; for example: for a retrospective study ethics application with positive approval: three pages; must be reviewed and approved by the institutional data protection coordinator; the time for the data coordinator is not included into the analysis; additional modifications may be required). In total, this adds up to 17 required pages.

### Costs for submitting

It was assumed that the time required to create and submit the ethics approval form is 4–10 hours, depending on the extent of the study and the person submitting it. Considering the hourly loan for a physician resident, the amount is approximately 130–325 €, depending on the resident’s level of expertise. Furthermore, 31.6–63.1 € must be added for approval and the head of the department’s signature, bringing the total to 161.6–388.1 €, assuming a first-year resident submits ethics approval forms. For a specialist physician, submitting costs can add up to 491.6 €. The details, including the time required and the total cost, are displayed in Table 1 (Tab. 1).

### Costs for the ethics approval committee

For the example case of a retrospective study comprising eight forms and 17 pages, Gemini assumed a total time needed for the ethics approval committee of 4.5–8.5 hours. Since it is hard to determine who serves explicitly on the ethics approval committee, the mean hourly wage of a senior consultant was used to estimate approximate costs. The details of the steps taken by the ethics committee, the time required, and the expenses incurred are displayed in Table 2 (Tab. 2). In total, the process from preparation of the ethics approval to the final vote takes about 8.5–18.5 hours, thereby incurring costs of approximately 403.3–844.4 €.

### Duplicate information

Due to the use of eight different forms, the physician preparing the ethics approval must enter duplicate information manually. In total, 32 duplications occurred across the forms. Duplications are displayed in Table 3 (Tab. 3).

## Discussion

In general, ethics approval for retrospective studies is an important part of ensuring good scientific practice. Therefore, our society should be aware of the costs associated with this and be willing to pay for good scientific practice.

Nevertheless, ethics approval for retrospective studies should be straightforward, as the risk to patients’ safety is minimal and it is not possible to retrospectively infer individual patient data from the published information. No additional information will be collected from the patient, and no further examinations or questionnaires will be administered (as in prospective studies). To the best of the authors’ knowledge, the formal requirements for ethical approval are only marginally more extensive for a prospective study. Only studies that involve additional radiation exposure, novel medical devices, or new drugs for patients are subject to more stringent ethical approval requirements; however, these studies also carry a significantly higher risk for participating patients.

To date, the ethics approval process for retrospective studies requires too many forms and documents, making it expensive. Considering that nearly every retrospective study requires ethics approval, there is significant potential for optimization.

Especially duplicate information required in submission forms could be avoided by summarizing it. Given the continued advancement of digitalization, it is completely outdated to require applicants to repeatedly provide the title, date, and department of a study project up to seven times during the application process. This problem is due to the pseudo-digital workflow of the approval process. For example, forms that require signatures must be printed, signed, then scanned and uploaded again. Furthermore, the authors find it unclear why the department head’s signature is mandatory.

The study is just an approximation of the real costs, since it is difficult to determine the exact number of time required and the associated costs, as many people are involved in the process to varying degrees. However, to the best of the authors’ knowledge, it is the only study to name the costs of ethics approval for retrospective studies. It is notable that, even when calculating conservatively, the costs of ethics approval for a retrospective study may amount to 844 € and take more than two days of work. This calculation is based on conservative approximations to reduce the risk of overestimating costs. 

For clarity, it should be noted that ethics approval for MHH-internal projects that are not externally financed is free of charge; the cost of this approval is nevertheless borne by society. However, employees involved in the application and review process cannot pursue their true vocation, such as patient care. For projects that are not MHH internal or publicly funded, a fee of 3,000 € is charged (first consultation, monocentric study) [13].

Medicine is always a risk calculation and must always consider the pros and cons. In this case, ethics approval for retrospective studies should not be omitted; rather, it should be streamlined to reduce the time required and the costs of creating and voting.

## Notes

### Author’s ORCID

- Max Lukas Linderkamp: 0009-0004-6703-4480

### Author contributions

M.L.L. and E.P. contributed equally. All authors contributed to the study conception and design. Material preparation and data collection were performed by M.L.L. and E.P., who also performed the analysis. M.-T.N. wrote the first draft of the manuscript, and P.K. and all authors commented on previous versions. All authors read and approved the final manuscript.

### Ethics approval

Since this study does not include patients’ data, no ethics approval is required.

### Data availability

The data that support the findings of this study are not openly available and are available from the corresponding author upon reasonable request. Data are stored in controlled-access data storage at MHH.

### Competing interests

The authors, Nils-Claudius Gellrich and Philippe Korn, receive speaker’s honoraria from KLS Martin-Group, Tuttlingen, Germany; DePuy Synthes^®^, Raynham, MA, USA; Straumann, Basel, Switzerland; and Brainlab, Munich, Germany.

## Figures and Tables

**Table 1:**
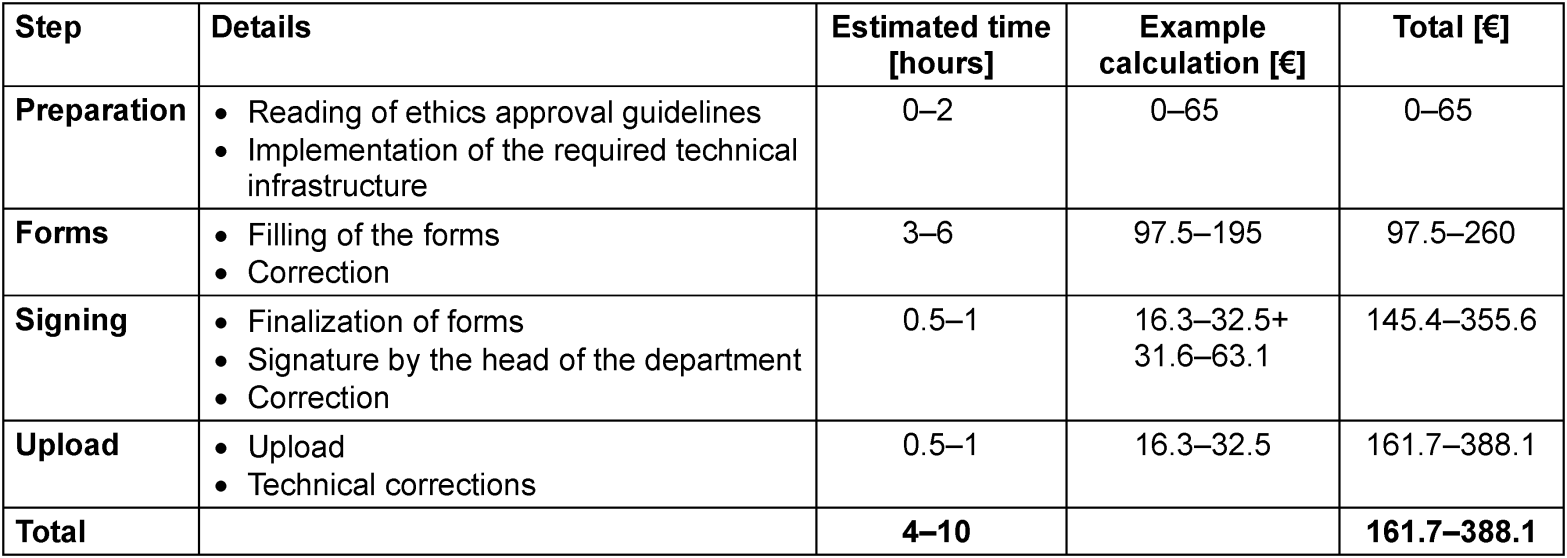
The table outlines the required steps and the details for submitting ethical approval. On the right are the estimated time and costs for the step, summarized in the right column. The example calculation assumes an hourly loan for a physician resident at the lowest level of expertise. Costs are rounded.

**Table 2:**
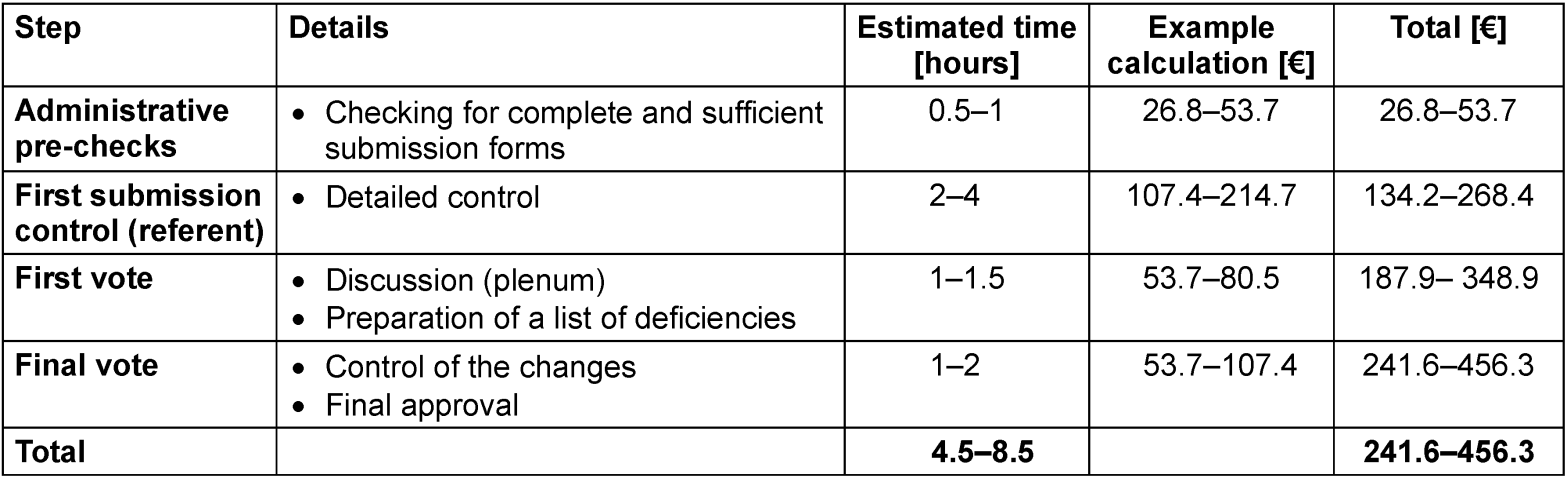
The table shows the steps taken by the ethics committee to control the submitted form, the estimated time required, and the approximate costs. The example calculation assumes an hourly loan for a senior consultant at the lowest level of expertise. Costs are rounded.

**Table 3:**
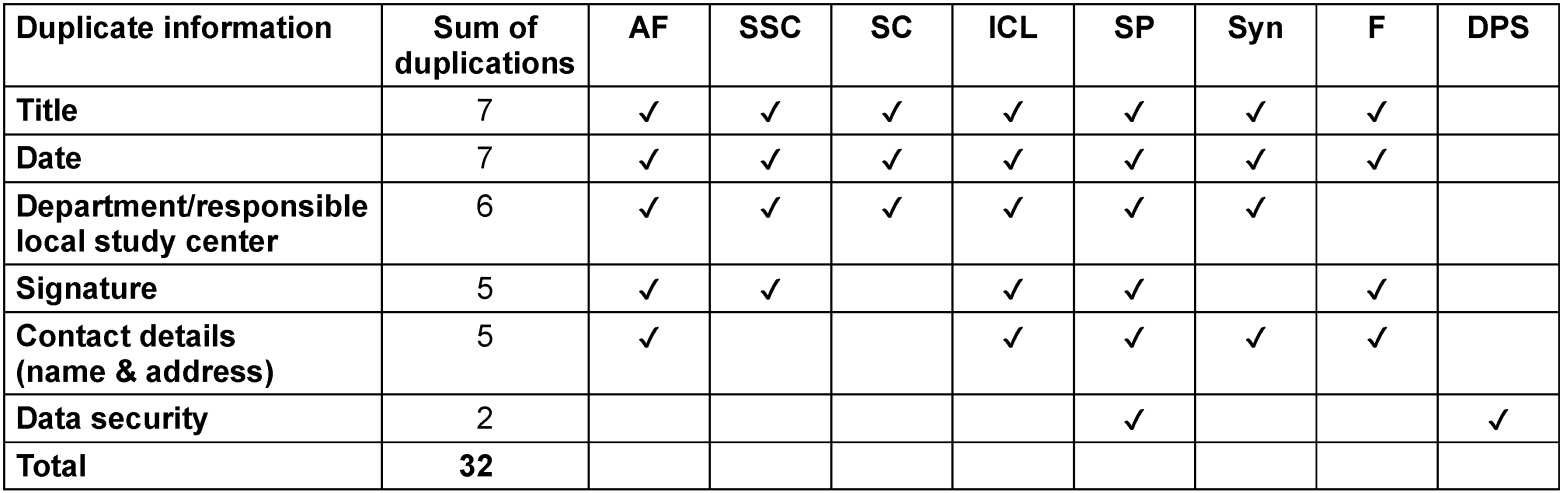
The table displays required duplicate information, the total number of duplications, and which forms require it.
